# Applications and limitations of machine learning in radiation oncology

**DOI:** 10.1259/bjr.20190001

**Published:** 2019-06-03

**Authors:** Daniel Jarrett, Eleanor Stride, Katherine Vallis, Mark J. Gooding

**Affiliations:** 1 Department of Engineering Science, Institute of Biomedical Engineering, University of Oxford, UK; 2 Mirada Medical Ltd, Oxford, UK; 3 Department of Oncology, Oxford Institute for Radiation Oncology, University of Oxford, UK

## Abstract

Machine learning approaches to problem-solving are growing rapidly within healthcare, and radiation oncology is no exception. With the burgeoning interest in machine learning comes the significant risk of misaligned expectations as to what it can and cannot accomplish. This paper evaluates the role of machine learning and the problems it solves within the context of current clinical challenges in radiation oncology. The role of learning algorithms within the workflow for external beam radiation therapy are surveyed, considering simulation imaging, multimodal fusion, image segmentation, treatment planning, quality assurance, and treatment delivery and adaptation. For each aspect, the clinical challenges faced, the learning algorithms proposed, and the successes and limitations of various approaches are analyzed. It is observed that machine learning has largely thrived on reproducibly mimicking conventional human-driven solutions with more efficiency and consistency. On the other hand, since algorithms are generally trained using expert opinion as ground truth, machine learning is of limited utility where problems or ground truths are not well-defined, or if suitable measures of correctness are not available. As a result, machines may excel at replicating, automating and standardizing human behaviour on manual chores, meanwhile the conceptual clinical challenges relating to definition, evaluation, and judgement remain in the realm of human intelligence and insight.

## Introduction

**Figure 1.**
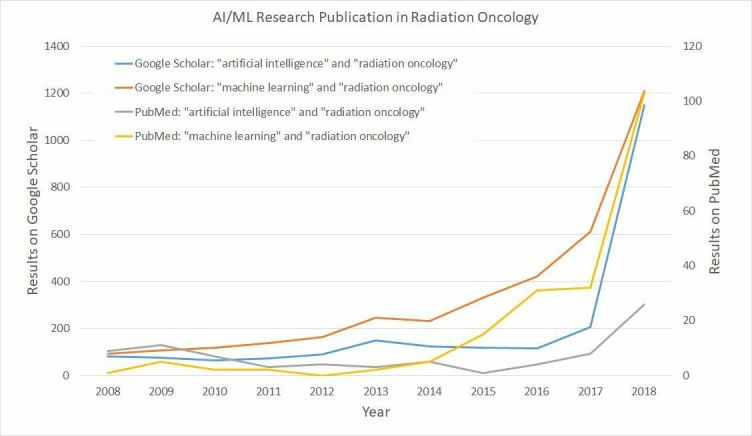
Number of search results by year for publications relating to “Radiation Oncology” and “Artificial Intelligence” or “Machine Learning”. Results from Google Scholar may represent a wider cross-section of publications than from PubMed. AI, Artificial Intelligence;ML, machine learning.

There is currently considerable enthusiasm for *Artificial Intelligence* (AI) in healthcare, including in radiation oncology. AI is an umbrella term covering all approaches to imitating human intelligence through the use of machines. However, the predominant technical approach currently generating interest in AI for healthcare is best categorized as *machine learning* (ML): the development of data-driven algorithms that learn to mimic human behaviour on the basis of prior example or experience. As a consequence, this paper focuses on the applications of ML in radiation oncology. The burgeoning interest in healthcare is evidenced by the rapid increase in clinical publications in this area, illustrated in Figure 1. Perhaps inevitably, one consequence of such enthusiasm in the evolving field is the risk of overblown expectations. Several reviews have surveyed the use of AI or ML in radiation oncology,^1–5^ or more broadly in healthcare.^6,7^ In order to calibrate our expectations, this paper first considers what the current clinical challenges are in radiation oncology and then assesses how, or whether ML, is addressing these challenges. With this clinical orientation, we do not describe algorithmic innovations in detail, and instead refer the interested reader to the more technically focused survey paper of Meyer et al^4^ or the book by El Naqa et al.^8^

**Figure 2.**
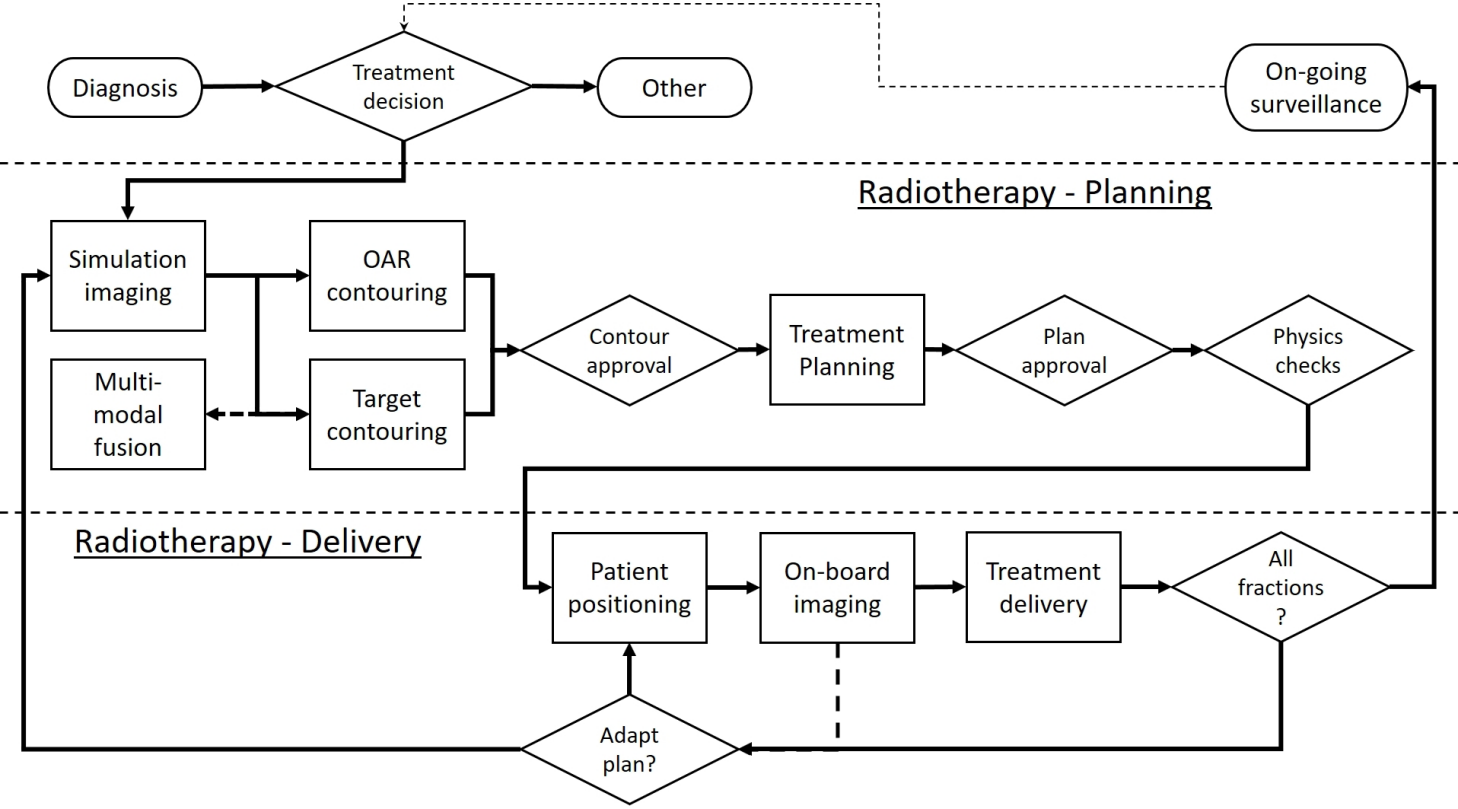
Schematic overview of the external beam radiation therapy workflow. Conceptually, we split this into (1) diagnosis and decision support, (2) treatment planning, and (3) treatment delivery. OAR, organ at risk.

The clinical workflow for external beam radiation therapy (EBRT) is considered to provide focus on how ML is addressing clinical challenges in radiation oncology, although many of the challenges faced will also apply in other areas of radiation oncology such as brachytherapy. A generalized representation of this workflow is shown in Figure 2; the precise details may vary between institutions. The workflow can be split into three conceptual domains: (1) diagnosis and decision support, (2) treatment planning, and (3) treatment delivery. The process of diagnosis and treatment decision-making can be considered part of the broader oncology workflow, and therefore this review only briefly touches on this aspect; instead, attention is focused on the more concrete applications of ML to treatment planning and delivery.

## Machine learning in planning

### Simulation imaging

Simulation is typically based on either CT imaging, MRI, or both—in which case image fusion is used to combine the information. CT imaging has been the predominant approach due to its cost-effectiveness, speed of acquisition, high spatial resolution, and availability of machines.

#### CT simulation

CT simulation imaging has been standard practice in external beam radiation therapy since the 1980s.^9^ It might be assumed therefore that many of the challenges have been overcome. However, a number of areas for clinical improvement remain. For example; a CT image only provides a single snapshot of the tumour, introducing location uncertainty right at the beginning of the planning process^10^; increasing the number and types of images used for planning, such as four-dimensional CT, and treatment monitoring may introduce additional risks to the patient with respect to radiation dose.^11^ Furthermore, uncertainty also stems from gauging the extent of the tumour^10^; this is discussed in more detail in the section on contouring.

Broadly speaking, there is a trade-off between dose reduction and image quality enhancement, and the achievable balance depends on the specific reconstruction algorithm. Improvements in image reconstruction aim to push the frontier of this trade-off, so that similar quality images can be acquired with a lower dose, or better quality images for the same dose. Research on ML for reconstruction follows on from improvements in iterative reconstruction,^12^ and many incorporate the use of neural networks (NNs). The majority of work has a focused on low-dose CT imaging for diagnosis, where dose reduction is the goal, rather than radiotherapy planning or delivery, where improving image quality is of greater importance, with the 2016 AAPM Low-dose CT grand challenge being a driver for several publications.^13–15^ ML techniques can be broadly classified by how the learning algorithm fits into the reconstruction pipeline. *Image-to-image* ML approaches aim to denoise the CT image *after* reconstruction has already taken place.^13,15,16^ Other approaches employ ML *during* the reconstruction process, to learn the prior information to guide iterative reconstruction from the raw imaging data,^14,17,18^ a two-dimensional array containing raw X-ray projections through the patient known as a sinogram. In addition, some studies specifically focus on the challenge of reducing metal artefacts in CT reconstruction; similarly, these can be categorized by whether artefact reduction occurs post-reconstruction,^19^ or directly by improving the sinogram.^20^

With their focus on diagnostic imaging, all of these studies utilize the peak signal-to-noise ratio (PSNR) and structural similarity index (SSIM) to assess visual image quality based on comparison with some putative “ground truth” image such as a higher dose CT image with a large number of views. On these counts, technical innovations often produce improvements over standard filtered back-projection or iterative reconstruction algorithms. However, these are seldom evaluated within the context of the radiotherapy workflow. For instance, while Gjesteby et al^19^ motivate their work with the need for high image quality in proton therapy and show a 26% increase in peak signal-to-noise ratio for metal artefact reduction compared to conventional approaches, no impact on the derived electron density, or the corresponding radiation dosimetry, of these methods have been considered.

#### MRI simulation

While CT imaging is ubiquitous due to its low cost and high speed, an inherent limitation of CT imaging is the uncertainty of tumour location due to poor soft-tissue contrast. In some cases, this makes MRI more attractive for simulation. While MRI is increasingly used for planning and treatment monitoring, progress has focused on how methods developed for CT can be adapted to work with MRI. However, the use of MRI for simulation introduces a new obstacle: the electron density information is unavailable for calculation of the treatment plan—a challenge shared with the task of attenuation correction in PET/MR hybrid-imaging.^21^ Conventional algorithmic approaches have been developed to synthesize CT images from MRI,^22^ and more recently ML approaches have been considered. These learn from training sets of MR and CT images, and broadly fall into two categories: direct *encoder–decoder* networks that map an MRI to pseudo CT images,^23,24^ and *generative adversarial networks* (GANs) that pit image “generators” against “discriminators” to ensure that synthesised images are as realistic as possible.^21,25,26^ While the majority of studies evaluate methods based on the similarity of pseudo CT images to known CT images, Maspero et al^25^ demonstrated that MR-based GAN approaches can achieve dosimetric deviations of as little as 0.5% under realistic conditions—a level of accuracy comparable to tissue classification-based approaches.^27^

### Multimodal fusion

Image registration is predominantly used in radiotherapy to align supporting imaging to the planning CT, such that additional information in the former can be used for target contouring.^28^ Typically, finding the best transformation between source and target images is posed as an optimization problem on a case-by-case basis. Specifically, they aim to maximize some measure of *similarity* between the images, subject to pre-specified physical constraints. However, conventional methods are limited clinically on two fronts. First, there is a clinical desire to accurately and automatically quantify spatial error throughout the image to inform treatment margins,^29,30^ which is particularly challenging in homogenous regions.^31^ Second, a significant conceptual challenge is the fact that image content may change between scans (*i.e*. before/after surgery or tumour growth), violating the assumption of a simple voxelwise correspondence. While a few example of ML have been applied in the context of radiotherapy to date,^32,33^ further studies have investigated this approach for other medical image registration tasks.

ML approaches to the registration problem have primarily focused on solving narrower technical issues related to the optimization problem: defining more sophisticated measures of similarity between images, as well as accelerating the computational procedure. Generally, it is difficult for handcrafted statistical similarity metrics to be simultaneously sophisticated enough to capture complex correlations between modalities, while remaining general enough to be robust to different tissues and noise levels without significant fine-tuning. One solution is to *learn* an appropriate similarity metric from training data. Building on earlier ML approaches,^34,35^ NNs have been trained as binary classifiers to learn patchwise correspondences between images.^36,37^ In technical studies, these learned measures of similarity were found to outperform conventional metrics such as mutual information and cross-correlation—both in terms of the correctness of the metric itself (in terms of prediction errors),^37^ as well as its effectiveness when used within the image registration workflow (in terms of overlap measures).^36^ Similarly, encoder–decoder networks that learn hierarchical features as a pre-processing step have been shown to be advantageous both in computational efficiency and robustness to noise.^33^ Alternatively, learning algorithms can also be employed to first construct shared latent representations of anatomical structures across different modalities,^38^ or even to perform cross-modal image synthesis,^26^ effectively reducing the multimodal problem to a more tractable monomodal one, with promising improvements in performance.^32^ Such promising technical advances still require further validation in a clinical setting.

ML strategies have also focused on accelerating the optimisation process. A recent approach attempts to predict deformation parameters *directly* from appearances of image patches, with a 35x speed up being reported for deformable registration of MRI brain imaging.^39^ In fact, learning the transformation function directly obviates the need for iterative optimization—the procedure simply becomes a much more efficient matter of applying a function. Furthermore, the registration task has been cast in terms of *reinforcement learning* (RL), where an *artificial agent* is trained to choose sequences of actions that improve image alignment.^40^ It has also been formulated within a GAN framework, where the generator estimates transformation parameters while the discriminator evaluates the quality of those predictions.^41^

Currently, for the purpose of clinical use, ML methods have been trained and evaluated on the basis of historical registrations within clinical data sets. “Ground truth” registrations are therefore estimates of the underlying transformations, and depend on the specific protocols and algorithms used to produce them in the first place. An alternative approach to training would be to use digital phantoms, as has been proposed for commissioning of clinical systems.^42^ Such an approach enables the true registration to be known but would still requires the registration used to be clinically meaningful to train a registration method to generate clinically plausible registrations. Thus, while learning algorithms can be trained to imitate registration patterns from examples, they are limited by the clinical correctness and realism of those examples. Importantly, the open clinical challenges related to quantifying spatial errors or accounting for anatomical changes have not been addressed to date. Despite these limitations, there are considerable benefits to be had both in time- and memory-efficiency gains by using ML for image registration.

### Contouring

In standard contouring workflows, the segmentation of tumour regions and normal tissue is manually performed by clinical staff, normally on a slice-by-slice basis. As a result, the procedure is lengthy and subject to a high degree of interobserver variability—constituting one of the largest sources of uncertainty in treatment planning.^43,44^ ^45^ The earliest attempts at automatic segmentation—such as edge- and region-based methods—relied purely on the informational content of each image in question. Later methods began to incorporate prior knowledge, such as relative anatomical locations or expected size variations of organs, into the process. The approach most commonly found in clinical use is *Atlas-based* segmentation.^46,47^ First, registration techniques are used to match target images to one or more selected reference images. Then, ground-truth segmentations on the references are mapped onto the target. However, atlas-based methods are highly sensitive to the atlas-selection strategy,^48^ as well as the robustness of the—often time-consuming—registration itself.

**Figure 3.**
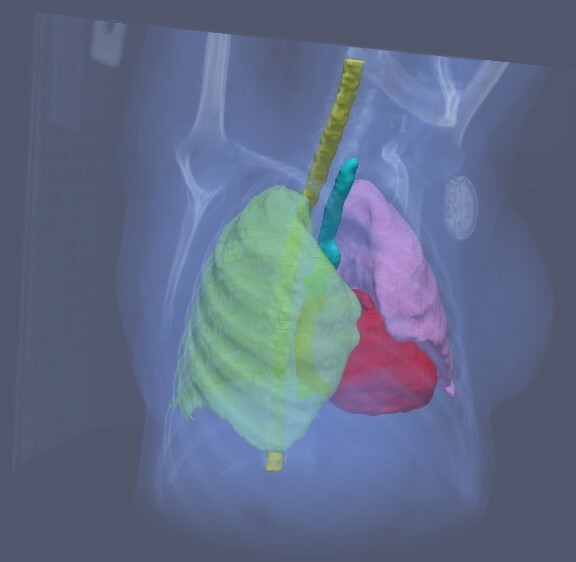
Example of unedited segmentation of OARs. The use of automatic OAR segmentation based on deep learning methods has demonstrated time savings in the clinical workflow. OAR,organ at risk.

The ML approach is to learn the structure labelling of each image voxel directly, more flexibly incorporating prior knowledge in the form of parameterised models. Successful techniques include the use of statistical and decision-learning classifiers,^49,50^ and more recently deep learning. For segmentation of organs at risk (OARs), *convolutional neural networks* (CNNs) have shown competitive performance in the context of thoracic cancer,^51^ head-and-neck cancer,^52^ prostate cancer,^53^ as well as more challenging organs such as the oesophagus.^54^ Although to date most research has focus on quantitative comparison against “ground truth”, Lustberg et al show an average time saving of 61% compared to existing clinical practice, and 22% compared to the use of atlas-based contouring.^51^ Figure 3 shows an example of unedited segmentation of OARs generated by a CNN. In addition, models have been shown to generalize successfully across imaging modalities without specific customisation or large volumes of data.^55^ The task of tumour volume segmentation is generally more challenging due to the varied shape, size, appearance, and localisation of tumours, as well as the lack of clear boundaries, rendering the process more reliant on oncologist knowledge and experience. Nevertheless, various deep learning architectures have shown considerable progress, for instance in brain cancer,^56^ breast cancer,^57^ oropharyngeal cancer,^58^ and rectal cancer.^59^

Contouring is inherently subjective,^44^ and highly variable as to what a “correct” result should look like. Learning algorithms are simply trained to maximize measures of similarity between their output and the examples given to them. So, while they are increasingly adept at mimicking human-drawn contours, they are constrained—as is any data-driven method—by the nature and quality of training examples. Machines can be no more “correct” than the human input taken as clinical ground truth^60^; until more concrete consensus definitions for object boundaries can be specified, machine “accuracy” is only meaningful within the context of individuals and institutional protocols. Ultimately, the fundamental challenge in generalizability lies in the standardization of existing clinical practices, without which it is difficult for any generic algorithm to perform equally well for different clinicians and centres. Nevertheless, the use of ML may assist in driving this standardization, enabling different institutions to start from a common reference and encouraging conformity to a standard.

Deep learning models have largely demonstrated superior quantitative performance relative to state-of-the-art algorithms for both target and OAR contouring,^52,56^ significantly decreasing the manual editing required for contours to be acceptable for use.^51^ Therefore, deep learning contouring approaches offers considerable potential to enable the automation and standardisation of contouring in clinical practice.

### Treatment planning

Radiation treatment planning is considered a problem of optimization—*i.e.* determining the parameters for positioning, fractionation, distribution, and other machine settings to best manage a patient’s cancer. However, the notion of *optimality* is often far from concrete in practice: in the presence of competing objectives for target coverage and organ sparing, the planning process requires multiple *subjective* trade-offs on the part of the oncologist and planner. As a result, conventional human-driven iterative workflows are often time-consuming and labour-intensive, with a high degree of variability.

Several planning strategies have been developed to address these issues. *Multicriteria optimization* generates a broad range of plans on the Pareto surface enabling the user to explore the impact of the trade-offs to be made in real-time.^61,62^ *Autoplanning* approaches mimic the process of iterative evaluations and adjustments made by experienced operators. They automatically identify regions of interest, appropriately introducing additional objectives and priorities within the traditional optimization procedure.^63,64^ *Knowledge-based planning* methods develop fixed associations between geometric and dosimetric parameters from a selection of previous plans. Rather than starting a new for each patient, this method leverages prior experience in predicting feasible dose–volume histograms^65^ or voxel dose distributions,^66^ navigating dosimetric trade-offs,^67^ or initializing optimization parameters to serve as individualized starting points for fine-tuning.^68^ Plans generated in this way have generally demonstrated similar coverage of target volume and superior OAR sparing compared to manually developed plans, as well as reduced planning time and interoperator variability in plan quality.^69,70^

Deep learning methods have recently been investigated for use in planning automation, predominantly focused on predicting a single solution as an input to knowledge-based or auto-planning or as a guide for the clinician/dosimetrist to predict achievable doses,^71^ rather than developing improved MCO methods. As a more dynamic and flexible incarnation of knowledge-based planning, NNs can be trained on prior plans to predict voxelwise dose values on the basis of contours and anatomy.^72,73^ In lieu of handcrafted features, CNNs are effective in automatic feature extraction, allowing models to learn contour-to-dose mappings directly.^71,74,75^ In the spirit of iterative autoplanning, GAN architectures have also been proposed to mimic the roles of dosimetrists (generators) and oncologists (discriminators).^76^ So far, deep learning methods have managed to produce highly accurate predictions of dose distributions,^72^ resulting in plans of equivalent or superior quality relative to historical plans—both in terms of meeting clinical satisfaction criteria, where GAN-based KBP outperformed the original clinical plans in meeting acceptance criteria in 75% of cases compared 73%,^76^ and in qualitative evaluation by clinicians where KBP using NN was preferred to the clinical plan in 81% of cases.^73^

While learning-based models are adept at incorporating human knowledge in planning and quality control, they are—once again—only as good as their training data. Importantly, there is a difference between generating an *optimal* plan, *vs* one that is simply *preferred* relative to its manual counterpart. Existing strategies have generally aimed—with much success—at the latter, broadly producing clinically acceptable and preferable results in head-to-head clinical evaluations. Although, subjective decisions requiring human judgement remain in determining the “best” compromise when not all constraints can be met or understanding how to vary the plan appropriately from “standard” plans for medically complicated cases, ML methods offer the potential to support this decision nevertheless, by accelerating the planning process and proposing high quality plans automatically.

### Approval & QA

Key components for plan evaluation and quality assurance include the delineation of target volumes and OARs, field arrangement, collimation, target coverage, dose homogeneity, and normal tissue sparing. Due to the increasing sophistication of radiation techniques, the potential scope of assessment has become correspondingly complex. Conventional workflows involve manual reviews with checklists and treatment charts—a process that becomes challenging with limited staff availability. Despite rigorous human review procedures, errors can and do slip through.^77^ Learning algorithms have the potential to automate elements of the process, model complexities without exhaustive rules and definitions, as well as enabling individualized assessments for more intelligent allocation of planning resources.

In terms of contour quality, ML algorithms can be trained to recognize and label anatomical structures within segmented images, using *confidence measures* to detect potential errors along the way.^78^ To validate physical parameters—such as beam configurations, monitor units, energies, and fractions—*anomaly detection* methods with statistical classifiers and clustering algorithms offer the ability to flag potential outliers for human intervention.^79,80^ As for dose distributions, knowledge-based approaches to predicting achievable doses have been effective for benchmarking the quality of planned dose distributions.^81–83^ In the same way as knowledge-based planning, libraries of historical plans are used to identify specific outliers for replanning, enabling a more targeted approach to improving suboptimal plans. Furthermore, ML algorithms can potentially be used to enhance the quality of delivery. For instance, it has been demonstrated that by predicting differentials between planned and delivered treatments variables, discrepancies can be incorporated into dose calculations, increasing γ passing rates during QA delivery.^84^ In addition, accurate a priori predictions of γ passing rates can be made on the basis of fluence maps and other plan details through probabilistic and deep learning techniques.^85,86^ However caveats apply: an algorithm cannot transcend its data and objectives. For instance, while ML may predict γ passing rates with great accuracy, it offers no help if passing rates do not actually correlate meaningfully with clinical safety.^87^

Importantly, great care must be taken to ensure that planning and validation algorithms are sufficiently independent—for instance, in the case of knowledge-based planning methods being used for quality assurance as well as autoplanning. After all, no matter how sophisticated an algorithm is, it cannot detect its own errors. Nevertheless, compared to generic checklist-based approaches, ML has the clear potential to augment the scope and efficiency of QA procedures via benchmarks and criteria tailored to specific patients, physicians, and institutions.

### Machine learning in treatment delivery

During the course of radiation therapy, treatment may need to be adjusted to ensure appropriate delivery of the plan. Adjustments may stem from both online factors such as the pre-treatment positioning of the patient, as well as longer term factors related to anatomical changes and treatment response. The technical challenges of aligning onboard imaging to the planning CT are similar to those discussed in the registration section—with the additional limitation that only restricted rigid adjustment can be performed, while the process of replanning requires additional imaging, contouring and treatment planning. An additional wrinkle, however, lies in determining which patients will most benefit from replanning—a decision that greatly influences the allocation of imaging and planning resources.

In terms of dose calculation for daily treatment adaptation, recent work has demonstrated promising gains in both efficiency and output quality by using NNs for directly mapping cone beam CT to planning CT images,^88,89^ where the use of GAN’s to correct CBCT values reduce mean intensity absolute errors compared to an original CT from 158HU to 57HU.^89^ During treatment delivery, the precision of irradiation may be compromised by respiratory motion. Learning techniques are particularly suited to capturing the heterogeneous variations in breathing patterns without explicit biomechanical models. Basic NNs have been shown to be effective in inferring and predicting tumour location from measurements of respiratory motion, thereby enabling adaptive beam realignment to occur in real time with minimal latency.^90^ In addition, learning frameworks combining individual predictors have been shown to significantly improve performance beyond the best existing methods.^91^

If the decision for plan adaptation is made during the course of treatment, knowledge-based methods can be adapted for automatic replanning, by initializing the planning procedure with the existing plan for the same patient.^92^ Recently, *reinforcement learning* (RL) approaches to automated dose-fractionation adaptation have been developed. In this framework, an artificial agent navigates a model of the radiotherapy environment, selecting sequences of planning decisions to maximise measures of reward—such as tumour control probability and normal tissue complication probability.^93^ More generally, RL algorithms can be flexibly trained to first *learn* representations of the radiotherapy state space, and then to *optimize* measures of treatment outcome on the basis of diagnostic, dosimetric, biological, and genetic features.^94^ By incorporating prior knowledge and clinical protocols via appropriate reward functions, as well as augmenting historical plans with adversarially generated synthetic data, this technique has been shown to achieve results comparable to those chosen by clinicians.^95^

ML has also been developed to identify candidate patients for replanning intervention. Based on anatomical and dosimetric variations (such as those caused by tumour shrinkage, organ movement, or changes in setup), classifiers and clustering algorithms have been developed to automatically predict patients who would most benefit from updated plans during fractionated radiotherapy treatment.^96,97^ However, since learning proceeds from data on historical patients, plans, and their adaptations, the limitation is—once again—that the algorithm is simply learning to mimic past prescriptions and protocols, instead of determining the truly ideal time for replanning intervention on the basis of outcomes.

Despite the caveats, the additional automation and efficiency that ML could bring to the radiotherapy workflow in registration, contouring and planning means that the cost associated with replanning is reduced. Thus, ML may allow the decision to replanning to be made with a greater focus on the clinical benefit, than on the cost of doing so.

## Discussion

For an ML algorithm to be effective, three ingredients are required: (1) a well-defined problem, (2) a well-defined ground truth for which there is sufficient data, and (3) a quantitative measure with which the algorithm is trained and evaluated. In the context of these requirements,

Table 1 summarizes the clinical challenges in radiotherapy, as well as the areas that ML research has focused on. The three requirements pose challenges to developing ML solutions at each step of the radiotherapy workflow and give insight into why some problems are readily addressed by ML, while others remain open problems.

**Table 1.** Summary of current ML research focus in the radiotherapy pathway

**Clinical application**	**Clinical Need**	**Current ML focus**	**Well-defined problem?**	**Well-defined ground truth?**	**Quantitative measure of correctness?**
CT simulation	Image reconstruction quality / dose reduction	Image reconstruction quality / dose reduction	Yes	No	No
MRI simulation	Pseudo CT creation	Pseudo CT creation	Yes	No	Yes
Image fusion	Estimate spatial uncertainty Accommodation of anatomical changes	Registration efficiency Appropriate similarity metric	No - Depends on use-case	No	No
Contouring	OAR/Target Contouring efficiency OAR/Target consistency Target contouring accuracy	OAR/Target Contouring efficiency OAR/Target consistency	Yes	No – Subjective clinical contours used	Yes
Treatment planning	Planning efficiency Plan consistency Determining the plan to deliver the best clinical outcome	Planning efficiency Plan consistency	No – Depends on clinical satisfaction criteria	No – Subjective treatment plans used	No
QA	Efficiency and automation Identification of clinically meaningful errors	Efficiency and automation	n/a	n/a	n/a
Delivery	Dose accuracy in the presence of motion (see Image fusion, Contouring, and Treatment planning) Determining who will most benefit from replanning	Dose accuracy in the presence of motion (see Image fusion, Contouring, and Treatment planning)	No	No	No

ML,machine learning; OAR, organ at risk; QA, quality assurance.

Training machine learning requires a well-defined problem, with a well-defined ground truth, and a simple measure with which to assess effectiveness. The application to QA is not considered in detail, as the status depends on what is being assured and to what degree.

First, is the problem well-defined? In other words, is there a *correct* answer in theory? For many clinical problems the answer is yes, but this is not always the case. In treatment planning, for instance, there is no clearly defined criterion for correctness. While a “good” plan can be conceived of as one on the Pareto optimal surface, there are no established guidelines regarding the choice of a single “best” point on that surface—especially since the “best” clinical outcome is analogously poorly defined.

Second, if there is a correct answer, do we have practical *access* to this ground truth? Once the problem is clearly defined, the next requirement is for training data. While the radiotherapy workflow can provide a lot of data, not all of it has a well-defined ground truth. For example, in image registration there is ambiguity regarding the correct displacement field within homogeneous image regions.

Finally, can we define a concrete measure to *optimize*? ML techniques are often trained by optimizing one or more performance metrics. This may pose a problem, since an appropriate measure is not always definable. For example, an ideal registration approach, whether through AI or traditional algorithms, would minimize the real clinical/anatomical error. However, this error is largely unknown (although it can be approximated for specific locations if they are marked-up). As a result, optimization is performed on surrogate measures, which may not accurately reflect clinical/anatomical correctness.

Table 1 summarizes current research in applications of ML to the radiotherapy pathway. As a consequence of the challenges faced in defining the problem, the ground truth, or the measure. ML research in radiotherapy currently does not address some of the greater clinical challenges, since the challenges are not necessarily always in the technical details of data analysis, but in defining either the question, the type of answer, or the way to measure how well we are doing. What is the correct registration for dose summation where tissues have changed? What is the clinical extent of the tumour on this CT scan? Which plan will deliver the best clinical outcome for this patient? How should we go about measuring this? These are problems that cannot be addressed by ML, but rather require the real intelligence and insight of humans.

Nevertheless, ML has seen considerable success in providing fast and parallelizable technical implementations to automate conventional workflows. While ML is not able to provide better definitions of the problem, application of ML in radiotherapy can bring efficiency and consistency to any solution to a problem. Increasing efficiency offers the opportunity to free time to consider the open clinical challenges, while increasing consistency has the potential to allow better assessment of the impact of intentional changes to treatment practice. By mimicking the current state-of-the-art reliably, ML could facilitate both transfer of best-practice between clinics and greater process automation.
